# Increased Prevalence of Psychiatric Conditions in Panniculectomy Patients With Prior Bariatric Surgery: A Nationwide Epic Cosmos Study

**DOI:** 10.7759/cureus.77321

**Published:** 2025-01-12

**Authors:** Gregory R Vance, Caleb S Bloodworth, Parker E Gleason, Katherine C Benedict, Seth J Kalin, Jared M Davis

**Affiliations:** 1 Division of Plastic and Reconstructive Surgery, University of Mississippi Medical Center, Jackson, USA; 2 Department of Plastic Surgery, MD Anderson Cancer Center, Houston, USA; 3 Department of Psychiatry, University of Mississippi Medical Center, Jackson, USA

**Keywords:** abdominoplasty, anxiety, bariatric, body, depression, dysmorphic, panniculectomy, psychiatric

## Abstract

Introduction

Patients with established body dysmorphic disorder (BDD) diagnoses are highly likely to be dissatisfied with plastic surgery and may have increased complication rates. Identifying at-risk patients prior to surgery is crucial to achieving patient satisfaction and minimizing adverse effects. This study seeks to assess the prevalence of BDD and associated psychiatric disorders in patients who have undergone panniculectomy and to evaluate potential associations between psychiatric comorbidity and prior bariatric surgery.

Materials and methods

Data used in this study came from Epic Cosmos (Epic Systems Corporation, Verona, WI, USA), a community collaboration of health systems representing over 233,000,000 patient records from over 1,325 hospitals and 28,900 clinics. All patients at least 18 years of age with an encounter between January 11, 2014, and January 10, 2024 (n=232,933,561) were included, and records were grouped based on procedural history using documented current procedural terminology codes. Prevalence of psychiatric diagnoses was measured using documented International Classification of Diseases (ICD) 10 codes, and 99% confidence intervals were recorded. Odds ratios (OR) were calculated for group comparison, and p<0.01 was used to determine significance.

Results

Patients who have undergone panniculectomy (n=62,671) demonstrated significantly increased prevalence of BDD (0.182% vs. 0.011%; OR=16.3, p<0.001), anxiety (43.2% vs. 13.5%; OR=4.9 p<0.001), and depression (35% vs. 9.7%; OR=5.0, p<0.001) compared to those who have not undergone the procedure (n=232,870,890).

Of the 62,671 patients with a recorded panniculectomy, those who had also undergone bariatric surgery (n=7,313) showed a significantly increased prevalence of BDD (0.397% vs. 0.154%; OR=2.6, p<0.001), anxiety (64.1% vs. 40.5%; OR=2.6, p<0.001), and depression (57% vs. 32.1%; OR=2.8, p<0.001) than those without prior bariatric surgery (n=55,358).

Conclusion

Patients undergoing panniculectomy are at high risk for BDD and other psychiatric comorbidities that justify formal screening prior to scheduling surgery. Surgeons should maintain a low threshold in seeking psychiatric evaluation for any concerning patients presenting for panniculectomy evaluation, especially in those with a bariatric surgery history, to ensure holistic benefit for their patients. Additionally, psychiatric professionals who understand the stress of elective plastic surgery should be utilized when available.

## Introduction

Body dysmorphic disorder (BDD) is characterized by the Diagnostic and Statistical Manual of Mental Disorders (5th ed.; DSM-5) as a preoccupation with a perceived defect in physical appearance that is either not observable or appears slight to others [1]. Individuals suffering from BDD will have significant distress or impairment in social, occupational, or other important areas of functioning. The DSM-5 also associates BDD with obsessive-compulsive disorder due to the “increasing evidence of these disorders’ relatedness in terms of a range of diagnostic validators as well as the clinical utility of grouping these disorders in the same chapter” [1]. While first-line therapy for these conditions is largely oriented toward supportive psychotherapy and cognitive behavioral therapy (CBT) [2], some patients seek to resolve their distress utilizing plastic surgery. It is important to recognize at-risk patients, as surgery does not address the underlying psychiatric stressor, potentially leading to higher rates of postoperative dissatisfaction and complication [3-4].

While the general population has an estimated BDD prevalence of one to three percent [5-6], multiple studies estimate that this prevalence increases drastically to 40-60% among patients undergoing aesthetic operations, such as body contouring surgeries, rhinoplasty, and abdominoplasty [7-9]. Interestingly, BDD patients who undergo these procedures often exhibit increased rates of postoperative dissatisfaction, potential exacerbation of the underlying psychological condition, and potential litigation [10-11]. The aesthetic surgery literature suggests that for operations such as abdominoplasty, more than 90% of patients with comorbid BDD are unsatisfied with their results [10].

Both reconstructive and aesthetic procedures are common after bariatric surgery and massive weight loss, more frequently for an abdominal panniculus than for other anatomic sites [12-13]. Ninety percent of post-bariatric patients report functional impairment related to abdominal panniculus [10]. The excess skin has been associated with intertriginous rash, infection, ulceration, and impaired mobility, which may indicate a panniculectomy to remove the panniculus [10]. While the procedure's primary purpose is relief from functional impairment, it is important to consider the role of aesthetic improvement in patient satisfaction.

Surgeons should be careful to ensure that this aesthetic benefit is not the primary concern of patients seeking panniculectomy. They should also evaluate for disproportionate perceptions of their truncal appearance in patients desiring abdominoplasty. Post-bariatric surgery patients desiring body contouring surgery have been shown to exhibit lower body image satisfaction scales and more depressive symptoms than their counterparts who were not interested in body contouring [14] and have shown an increased prevalence of lifetime major depression, panic disorder, generalized anxiety disorder, and BDD [15]. In this population, performing a primarily functional surgery when the patient expects a primarily aesthetic outcome may lead to increased dissatisfaction in the setting of BDD.

The current study seeks to assess the prevalence of BDD and associated psychiatric disorders in patients who have undergone panniculectomy and to evaluate potential associations between psychiatric comorbidity and prior bariatric surgery, with the hope of more completely characterizing this patient population on a nationwide scale. Hypotheses include an increased prevalence of psychiatric comorbidity in those who have undergone panniculectomy and a compounded effect on this association with a concurrent history of bariatric surgery. This study hopes to describe further the incidence of psychiatric comorbidity in these patient populations and to highlight the importance of preoperative screening and optimization prior to panniculectomy when indicated.

This article was previously presented as an oral presentation at the 2024 SESPRS 67th Annual Virtual Medical Student Session on April 26, 2024.

## Materials and methods

Data used in this study came from Epic Cosmos (Epic Systems Corporation, Verona, WI, USA), a community collaboration of health systems representing over 233,000,000 patient records from over 1,325 hospitals and 28,900 clinics. All adult patients encountered between January 11, 2014, and January 10, 2024, were included in the patient population. Included patients had a recorded age of at least 18 at the time of their panniculectomy or at the time of their recorded encounter if they had not undergone the procedure.

Patients were first divided into groups based on the history of panniculectomy, using the inclusion and exclusion of current procedural terminology (CPT) code 15380 in the SplicerDicer feature of Epic Cosmos. Within each of these groups, the prevalence of BDD, anxiety disorders, and major depressive disorders was recorded using respective ICD-10 codes. Then, the group with a history of panniculectomy was further divided by history of bariatric surgery, using 19 distinct CPT codes for inclusion and exclusion of bariatric surgery. Documentation of any of these codes included the group with a bariatric surgery history. The same ICD-10 codes were used to record the prevalence of BDD, anxiety disorders, and major depressive disorders. Utilized CPT and ICD-10 codes with respective sample sizes can be found in Table 1.

**Table 1:** Grouping by CPT and ICD-10 codes with associated population size CPT: current procedural terminology, ICD: International Classification of Diseases, BDD: body dysmorphic disorder

Procedure	CPT code(s)	n
Panniculectomy	15830	62,671
Panniculectomy with bariatric surgery	43644, 43645, 43659, 43770-43775, 43842-43848, 43886-43888, 43999	7313
Panniculectomy without bariatric surgery	-----	55,358
No panniculectomy	-----	232,870,890
Diagnosis	ICD-10 code(s)	n
BDD	F45.22	26,199
Anxiety	F41, F41.0, F41.1, F41.3, F41.8, F41.9	26,148,828
Depression	F32, F32.*, F32.A, F32.0, F32.1, F32.2, F32.3, F32.4, F32.5, F32.9	18,672,832

The prevalence of each recorded psychiatric diagnosis, or group of diagnoses, was recorded for each group with a 99% confidence interval. Odds ratios (OR) were calculated for group comparison. Standard normal deviations (z-values) were calculated as the natural log of an OR divided by the standard error of the natural log of the respective OR. The p-value is defined as the area of the normal distribution that falls outside the positive or negative z-value. Significance was determined by p<0.01.

## Results

**Figure 1:**
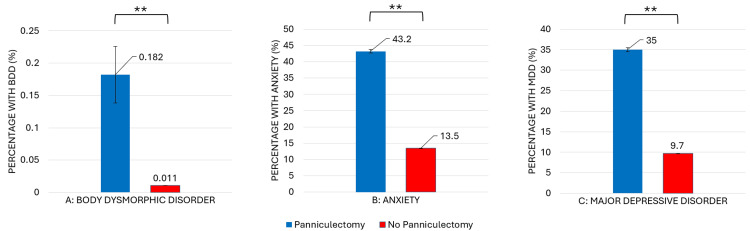
Relative prevalence of psychiatric comorbidities in adults with and without panniculectomy history

Comparing adults with and without a history of panniculectomy, undergoing the procedure was significantly associated with increased prevalence of each group of psychiatric diagnoses studied. As shown by Figure 1 and Table 2, those who had undergone panniculectomy were over 16 times more likely to have a documented diagnosis of BDD and over five times more likely to have documented anxiety- and depression-related diagnoses.

**Table 2:** Comparing prevalence of psychiatric diagnoses in adults with and without a history of panniculectomy The natural log (ln) and SE of the OR were calculated. A standard normal deviation (z-value) was calculated as ln(OR)/SE{ln(OR)}, and the p-value is the area of the normal distribution that falls outside ±z. OR: standard ratio, SE: standard error, BDD: body dysmorphic disorder

	Prevalence	OR	Z-value	p-value
BDD				
Panniculectomy	0.182% ± 0.044%	16.267	29.688	<0.001*
No panniculectomy	0.011% ± 0.001%			
Anxiety				
Panniculectomy	43.2% ± 0.509%	4.904	197.171	<0.001*
No panniculectomy	13.5% ± 0.006%			
Depression				
Panniculectomy	35.0% ± 0.491%	5.028	192.775	<0.001*
No panniculectomy	9.7% ± 0.005%			

**Figure 2:**
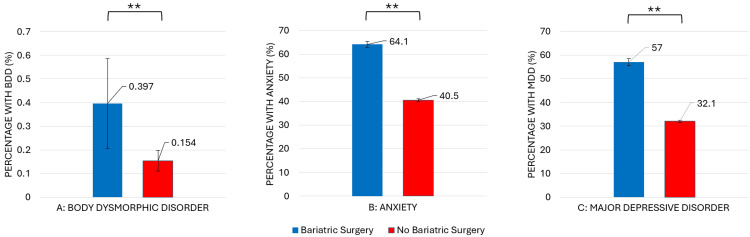
Relative prevalence of psychiatric comorbidities among panniculectomy patients with and without prior bariatric surgery

When dividing panniculectomy patients based on the presence or absence of bariatric surgery history, documented bariatric surgery was also significantly associated with increased prevalence of BDD, anxiety, and depression, as shown in Figure 2 and Table 3. Each of these categories of psychiatric conditions was over two times more likely to be documented in those with documented bariatric surgery.

**Table 3:** Comparing the rate of psychiatric diagnosis in panniculectomy patients with and without documented bariatric surgery history The natural log (ln) and standard error (SE) of the OR were calculated. A standard normal deviation (z-value) was calculated as ln(OR)/SE{ln(OR)}, and the p-value is the area of the normal distribution that falls outside ±z. OR: odds ratio, SE: standard error, BDD: body dysmorphic disorder

	Prevalence	OR	Z-value	p-value
BDD				
Bariatric surgery	0.397% ± 0.189%	2.589	4.416	<0.001*
No bariatric surgery	0.154% ± 0.043%			
Anxiety				
Bariatric surgery	64.1% ± 1.4%	2.625	37.307	<0.001*
No bariatric surgery	40.5% ± 0.537%			
Depression				
Bariatric surgery	57.0% ± 1.5%	2.797	40.640	<0.001*
No bariatric surgery	32.1% ± 0.511%			

## Discussion

BDD is a severe psychiatric disorder characterized by obsessions about perceived physical deficits, often leading to distress and impairment in regular activities. It may be clinically challenging for plastic surgeons to differentiate these patients from those with significant functional impairment due to anatomic variables, which is crucial. Although surgery can successfully alter appearance, it cannot address the psychiatric manifestations of BDD, which can lead to exacerbation of psychiatric illness, postoperative distress, and patient dissatisfaction. Further, positive body image may be more vital in maintaining mental health than actual physical appearance [16], highlighting the importance of proper psychiatric treatment in patients with BDD.

While there is undoubtedly an aesthetic component to panniculectomy, it does not seek the same primary outcome as cosmetic abdominoplasty, and the importance of ensuring that patients understand this distinction cannot be overstated. Panniculectomy aims to reduce complications such as dermatologic infections and mobility impairments secondary to an abdominal panniculus. While the primary goals are functional, panniculectomy can improve physical appearance, and patients may have difficulty distinguishing between panniculectomy and abdominoplasty regarding the expected aesthetic outcome.

Our study demonstrates that patients undergoing panniculectomy are at a much higher risk for BDD than the general population, particularly those with a history of prior bariatric surgery. With a 16-fold increased risk of a BDD diagnosis in this population, those presenting in evaluation for panniculectomy should be screened appropriately prior to surgery using tools with proven utility such as the Body Dysmorphic Disorder Questionnaire, the Body Dysmorphic Symptom Scale, or the Body Dysmorphic Disorder Screener for DSM-5 [17-19]. Additionally, in comparison to prior prospective studies that utilize these screening tools that suggest a highly elevated BDD prevalence in those seeking aesthetic operations [7-9], the overall prevalence of documented BDD diagnosis that we found suggests a massive level of underdiagnosis of the condition in this population. This should also encourage extensive and intentional history-taking and evaluation on the part of the physician.

With a functional outcome as the primary goal, it is important to recognize numerous factors impacting postoperative outcomes in panniculus removal procedures. Many patients with a history of bariatric surgery have increased rates of comorbid conditions that can result in unique medical profiles and complication risk in the setting of abdominal contouring procedures [20]. While the investigation of medical comorbidities such as diabetes, sleep apnea, and hypertension prior to surgery has been widely recommended [21-23], the data presented suggests that a similar emphasis on psychiatric comorbidities is equally essential. These findings underscore the multitude of psychiatric risks in this population and the necessity of preoperative assessment.

Due to this pronounced psychiatric comorbidity risk, surgeons should be vigilant and have a low threshold for professional psychiatric referral, especially for those with a known bariatric surgical history being evaluated for panniculectomy. Although one of the core weaknesses of preoperative assessment is its time-consuming nature [24], the patient's well-being should be prioritized over the pursuit of efficiency and financial gain. Short-term improvement in BDD symptoms and severity with CBT has been previously noted [25-26], but there is significant potential for long-term regression and stagnation of improvement [27]. Therefore, in the studied patient population, prolonged psychiatric evaluation and treatment may be required at the discretion of the psychiatric provider prior to further surgical intervention. In these patients, adequate management requires a strong relationship between specialized providers to provide robust multidisciplinary care when appropriate.

There are significant limitations to this study that warrant discussion. Because of the cross-sectional and retrospective nature of this analysis on this vast, heterogeneous population, we lacked the ability to evaluate the effects of demographic and clinical variables such as gender, age, and BMI on our findings. We could also not assess other organic causes of psychiatric presentation due to the study design. Additionally, we did not parse out the various methods of bariatric surgery, which we acknowledge may cause varying weight loss efficacy and thereby affect panniculus presentation and operation. The primary goal of this study was to evaluate psychiatric risk purely at the population level. Still, we believe that future prospective studies on this topic will be more amenable to variables of this nature.

Our findings on the relative prevalence of psychiatric conditions depend significantly on thorough psychiatric evaluation and accurate psychiatric diagnosis using diagnostic criteria outlined in the DSM-5. The accuracy of these values also depends heavily on physicians entering proper ICD-10 codes for diagnoses and CPT billing codes for completed procedures. Additionally, because of the deidentified nature of the Epic Cosmos database, many contextual variables surrounding patient psychiatric history are lost, such as condition time of onset and severity, specialty of diagnosing physician, and potential stressors. Because of this, any effects of pharmaceutical or cognitive psychiatric treatment and other confounding variables cannot be delineated and discussed.

## Conclusions

The results of this study emphasize the need for a multidisciplinary approach to patient care in panniculectomy evaluation, with mental and physical health evaluations as integral components of the surgical evaluation process. Extensive work should be done to continue to provide multidisciplinary healthcare teams with information about population demographics and outcomes regarding panniculectomy surgery in the setting of mental health comorbidities. Future research should focus on additional external risk factors specific to panniculectomy patients for comorbid psychiatric conditions. A prospective model studying patients being evaluated for panniculectomy would be the ideal scenario to this end, with psychiatric screening for BDD and other comorbid conditions as a primary focus. This opens the possibility of studying not only additional potential risk factors and comorbidities but also the effects of psychiatric referral and treatment on later complication rates.
